# Avian Respiratory Coinfection and Impact on Avian Influenza Pathogenicity in Domestic Poultry: Field and Experimental Findings

**DOI:** 10.3390/vetsci5010023

**Published:** 2018-02-24

**Authors:** Ahmed Samy, Mahmoud M. Naguib

**Affiliations:** 1 Reference Laboratory for Veterinary Quality Control on Poultry Production, Animal Health Research Institute, Dokki, Giza 12618, Egypt; 2 Zoonosis Science Centre, Department of Medical Biochemistry and Microbiology, Uppsala University, Box 582, 751 21 Uppsala, Sweden; 3 Infectious Medicine, Department of Medical Sciences, Uppsala University, 751 85 Uppsala, Sweden

**Keywords:** co-infection, avian influenza, poultry, secondary infection

## Abstract

The avian respiratory system hosts a wide range of commensal and potential pathogenic bacteria and/or viruses that interact with each other. Such interactions could be either synergistic or antagonistic, which subsequently determines the severity of the disease complex. The intensive rearing methods of poultry are responsible for the marked increase in avian respiratory diseases worldwide. The interaction between avian influenza with other pathogens can guarantee the continuous existence of other avian pathogens, which represents a global concern. A better understanding of the impact of the interaction between avian influenza virus and other avian respiratory pathogens provides a better insight into the respiratory disease complex in poultry and can lead to improved intervention strategies aimed at controlling virus spread.

## 1. Introduction

Avian influenza viruses (AIVs), caused by influenza A viruses, are members of the Orthomyxoviridae family [1]. AIVs infect both domestic poultry and wild birds; in addition, many reports have described their natural transmission to humans and occasionally to other mammals [2,3]. AIVs are further classified, based on the presence of multiple basic amino acids at the cleavage site of their hemagglutinin (HA) protein and/or their virulence in chickens, as low pathogenic avian influenza (LPAI) or high pathogenic avian influenza (HPAI) viruses [4]. 

LPAIVs continue to spread worldwide and have been isolated frequently from apparently normal migrating waterfowl, shorebirds, and domestic poultry [5]. LPAIV has become endemic in domestic poultry in different countries in Asia and the Middle-East, causing subclinical infections, mild respiratory symptoms, and/or drops in egg production [6]. Since 2003, HPAIV H5N1 of the Eurasian lineage has spread worldwide in a very short time, causing continuous emerging threats mainly to the Middle East and Asian countries [7,8]. Recently, frequent reassortments of HPAIV H5 have been noted, with other co-circulating AIVs in different countries in Europe, North America, East Asia, and the Middle East [9,10,11].

In poultry, HPAIV induces a very high mortality rate and a sharp decline in productivity both under field conditions and experimental trials using specific pathogen-free (SPF) chickens. In contrast, LPAIV induces minimal or no clinical signs in controlled SPF challenge experiments; however, field observations associated with LPAIV H9N2 infections showed slight mortality rates and a loss in egg productivity. One of the most important reasons for this difference is the mixed infection with other viral and/or bacterial respiratory pathogens. Mixed infections of LPAIV and/or HPAIV were reported in association with other viral diseases such as Newcastle disease virus (NDV) and/or infectious bronchitis disease (IB). In addition, many bacterial diseases were observed with positively AI-infected birds such as *Mycoplasma gallisepticum (*MG*), Mycoplasma synoviae (*MS*)*, *Ornithobacterium rhinotracheale (*ORT*)*, *Escherichia coli,* and *Staphylococcus*. The impact of such co-infections on the host responses, which includes viral shedding, sites of virus replication sites, and clinical outcome, was addressed in several studies [12,13,14,15,16] under field and experimental conditions.

The interaction between pathogens at the same infection site could be either synergistic or antagonistic, and these interactions could subsequently determine the severity of the disease complex. These two phenomena depend on interaction time (pre, simultaneous, or superinfection), host immune response, biological product, and/or other environmental factors. 

Viral respiratory infections in human, mammals, and avian species can augment the secondary bacterial infection, including commensal bacteria, through mechanical damage of ciliated and goblet cells. This in turn facilitates the bacterial attachment and colonization [17,18,19] or impairment of the phagocytic activity and/or the alteration of the innate immune response [20,21,22]. It is worth noting that the worst effect of the secondary bacterial infection is not only the exaggeration of the pathogenesis but also an increase in bacterial density [23]. On the other hand, bacterial preinfection could be of benefit to AIV pathogenesis as in cases of protease secreting bacteria that facilitate cleavage of the HA of LPAIV [24,25]. In contrast, bacterial infection might limit the viral pathogenicity either through augmentation of the immune response to viral pathogens and/or inhibit or reduce the viral attachment to susceptible cells [26]. 

The surface glycoprotein hemagglutinin (HA) represents the main determinant of the influenza A pathogenicity, as it initiates the infection and then mediates the fusion of viral and endosomal membranes. The HA protein of HAPIVs contains multiple basic amino acids at the cleavage site cleaved by ubiquitous expressed furin in the target cell cytoplasm leading to systemic infection [27], while LPAIV-HA contains monobasic amino acids at the cleavage site that are cleaved extracellularly by cell type-specific, extracellular trypsin-like proteases, leading to localized infection in respiratory and intestinal tissues [28]. The bacterial infection, mediating the proteolytic activation of AIV, has been well documented; however, the exact mechanism is still not well known. Four proposed mechanisms could illustrate the role of bacterial coinfection: (a) directly through the cleavage of the HA, (b) indirectly through activation of host proteases, (c) as an antagonist of the host protease inhibitors, and (d) as a stimulation of host inflammatory response that subsequently increases the leakage of host proteases [29,30,31]. The common recovery of highly proteolytic bacteria in the avian upper respiratory tract flora highlights the importance of respiratory bacterial proteases that mediate the proteolytic activation of AIVs [32].

Infection of a host with a heterologous virus may result in the occurrence of viral interference [33]. Viral interference is a phenomenon in which virus-infected cells do not permit the replication of a second homologous or heterologous virus [33,34]. Viral interference can be elucidated by different mechanisms encompassing (a) competition for cell receptors attachment for replication, (b) intracellular host machinery competition, and (c) virus-induced interferon interference. Measurable differences that are described as being associated with mixed virus infection include changes in viral replication patterns, tissue tropism, pathological responses, and immunological responses [34]. This viral interference can be detrimental by obtaining the correct complete diagnosis, since co-infected flocks revealed lower or undetectable virus titers that might mask the real diagnostic picture. Co-infection usually occurs, but due to confusing similar clinical signs it cannot be easily diagnosed. 

So far, most of the viral-viral or viral-bacterial interference studies do not entirely reflect the field situation in which poultry are exposed to more than one infectious and/or non-infectious agent. The aim of this review is to assess the incidence, clinical significance, and impact of coinfection/secondary infection associated with AI in different domestic poultry species.

## 2. Avian influenza and other viral co-infections

### 2.1. Co-Infection with LPAIV and HPAIV

The vast majority of AIVs are LP, causing mild respiratory signs or drops in egg production, while some birds display no clinical signs at all. HPAIV is a contagious infectious disease of poultry, producing necrosis, hemorrhage, and inflammation in multiple visceral organs, as well as in the brain and the skin [2].

Co-infections with both HPAIV and LPAIV were reported in many natural cases in different countries, such as co-infection of LPAIV H9N2 with HPAIV H5N1 in China, Israel, Bangladesh, and Egypt [35]. Reassortment can emerge when two different AIV subtypes co-infect the same cell at the same time. Several reassortment strains of AIV subtypes have been reported in different countries in Europe, North America, East Asia, and the Middle East [9,10,11]. In addition, experimental studies revealed that LPAIV H9N2 A/Chicken/HK/G9/97 plays a role in protecting chickens from a lethal challenge of a HPAIV H5N1 infection [36]. However, infected chickens shed HPAIV H5N1 in their feces (higher levels) and trachea (lower level), while the signs of the co-infected chickens were usually mild and included sneezing, nasal discharge, and ruffled feathers. It was suggested that chickens were protected against HPAIV H5N1 through the cross-reactive cellular immunity produced by LPAIV H9N2 influenza viruses. Another study, described by Khalenkov, et al. [37], reported that the chickens, previously inoculated with one of the Israeli LPAIV H9N2 genotype G1, could show up to 100% survival after inoculation with the lethal H5N1 virus. Both findings suggest that co-circulation of H9N2 with H5N1 can contribute to the perpetuation of lethal HPAIV H5N1. In addition, a recent study revealed that previous infection, not vaccination with LPAIV, modulates the course of subsequent infection with the Egyptian HPAIV H5N1 [38].

### 2.2. Avian Influenza and Infectious Bronchitis Co-Infection 

Infectious bronchitis (IB) is an acute rapidly spreading disease of chickens characterized by respiratory signs, drop in egg production, and poor egg quality or nephritis/nephrosis [39,40]. Numerous distinct genotypes have been reported in many countries worldwide [41]. IBV is considered one of the most economically important respiratory viral diseases, and it threatens the poultry industry worldwide, along with the HPAIV and velogenic Newcastle disease virus (vvNDV) [42]. IBV was described as being associated with both HPAIV and/or LPAIV as a natural infection in different countries in Asia and the Middle-East [15,43]. IBV initially infects the epithelial surface of the respiratory tract of chickens, then spreads via viremia in a range of other tissues for further replication (such as kidney, gastrointestinal tract, and oviduct) [40,44]. A trypsin-like serine protease domain, encoded by the open reading frame ORF1a of the IBV, has been reported; IBV infection may provide this enzyme and enhanced LPAIV H9N2 pathogenicity in chickens [45]. The literature on coinfections of AI virus with IB virus is sparse on co-infection with IB vaccine or IB field stain 4/91.

#### 2.2.1. H9N2 with IB Vaccine

The IBV vaccine is used extensively in chicken farms in many countries worldwide where both IBV and LPAIV H9N2 are endemic [43,46]. An experimental study with LPAIV H9N2 (A/chicken/Iran/SH-110/99(H9N2)) and an IBV live vaccine (H120 strain) was conducted concerning clinical signs, gross lesions, viral shedding, and mortality. It was reported that the interference between LPAIV H9N2 and the IBV live vaccine increased the severity of LPAIV H9N2 clinical signs (depression, ruffled feathers, respiratory distress (coughing, sneezing, and dyspnea), swelling of the periorbital tissues and sinuses, conjunctivitis, and nasal and ocular discharge), as well as the severity of gross lesions (such as tracheal congestion, lung hyperemia and exudation of the trachea with tubular cast formation in the tracheal bifurcation). In addition, this led to a prolonged shedding period of LPAIV H9N2 that extended from day 2 to day 11 compared to day 3 to day 9 in chickens infected only with LPAIV H9N2; it even caused death in the infected birds [14]. 

#### 2.2.2. LP H9N2 and IB Field Strain

To rule out the effect of the co-infection of LPAIV H9N2 and IBV field strain, an experimental study was performed in chickens by Seifi, et al. [47]. Chickens, co-infected simultaneously with LPAIV H9N2 (A/Chicken/Iran/SH110/99) and IBV (IBV/4/91), reported severe clinical signs (respiratory distress, facial edema, conjunctivitis, depression, lacrimation, ruffled feathers, whitish watery diarrhea, and nasal discharge which continued until eight days post-infection), gross lesions (tracheal congestion, air saculitis, lung hyperemia, tubular cast formation in the tracheal bifurcation which extended to the lower bronchi, swollen kidney, and hemorrhagic pancreas and intestine), and mortality rate (5%), which were significantly different when compared with chickens infected separately with the same virus. In addition, a significantly higher HI titer against AIV infection was noticed in the co-infected group, which may influence the diagnosis process of this virus in the field [47]. These findings showed that IBV co-infection enhanced LPAIV H9N2 pathogenicity through protease enzymes.

### 2.3. Avian Influenza and Newcastle Disease Virus Co-Infection 

The Newcastle disease virus (NDV) is a member of the genus Avulavirus in the *Paramyxoviridae* family [48]. NDV possesses different pathotypes based on the sequences of the protease cleavage site of the fusion (F) protein and virulence in chicken [49]. The original classification of NDV was based on its virulence, either as low virulence (lentogenic), moderately virulent (mesogenic), or virulent (velogenic) [48]. These viruses transmit from their natural reservoirs (wild birds) to domestic birds, initially producing subclinical infections, and occasionally causing upper respiratory disease and a decrease in egg production.

NDV and AIV are two of the most economically important viruses that affect poultry sectors worldwide [50]. Frequent co-infections/interferences were reported in many areas of the world, especially where both viruses are endemic in many forms, like (a) lentogenic NDV with either LPAIV or HPAIV and (b) velogenic NDV with either LPAIV or HPAIV [51]. 

NDV and AIV can replicate in the upper respiratory and intestinal epithelial cells by binding to the sialic acid-containing receptors on the cell surface (through the HN or HA protein of NDV or AI respectively) [52]. Virus replication may also be affected by the previous replication of another virus in the same site through the active antiviral immune responses including immunomodulators, induced interferon, or recruitment of immune cells [53]. The impact of such co-infections on several host responses including clinical signs, viral shedding, and gross lesion was addressed in various studies.

#### 2.3.1. lNDV and LPAI in Chicken

A co-infection study on LPAIV subtype H9N2 (A/Chicken/Pakistan/UDL/08) and lNDV vaccine strains (LaSota) in chicken showed a significant reduction in virus replication. Lower virus sheddings were observed in the first 3 days post infection as compared to singly inoculated chickens. Furthermore, significantly higher titer for LPAIV and lower for lNDV was detected in oropharyngeal and cloacal swabs, and no clinical signs were observed [54]. 

Another co-infection study on evolved LPAIV H7N2 (A/turkey/VA/SEP/67/2002) and lNDV (Lasota) was performed in chicken. No clinical signs were observed in co-infected chickens, and the same shedding pattern was observed as in the previous study [55].

#### 2.3.2. vVNDV and HPAI in Chicken

Natural co-infections with both HPAI and vNDV strains were reported in many countries, although both diseases are clinically indistinguishable [29]. An experimental co-infection model reported that the virulent NDV (velogenic CA/2002) interferes with the replication of HPAIV (A/chicken/Queretaro/14588-19/95(H5N2)). Reduction in the number of birds shedding HPAIV was observed. However, the death of all birds was recorded within 1.9 to 5.2 days with no difference in the clinical signs between the single infected or co-infected group in high dose of vNDV. However, it has been noted that a low dose of vNDV increased the survival of the co-infected chickens [56]. Conversely, chickens infected with the less virulent mesogenic NDV prior to HPAIV (with a lower dose) revealed reduced HPAIV replication and increased survival rates. In conclusion, viral interference depends on viruses’ titer, virulence, and time of infection [56]. 

#### 2.3.3. vNDV with Either a LPAIV or a HPAIV in Duck 

Co-infection and interference between AIV and vNDV was also demonstrated in ducks [57]. No clinical signs were observed in infected or co-infected ducks with vNDV (APMV-1/duck/Vietnam (Long Bien)/78/2002) and LPAIV (A/Mallard/OH/421/1987 H7N8). However, co-infection decreased the number of birds shedding vNDV, while it did not affect the number of ducks shedding LPAIV (except at 2 dpi low virus shedding was observed in the co-infected group than the group infected only with LPAI). Ducks simultaneously infected with vNDV and HPAI (A/duck/VN/NCVD-672/2011 (H5N1)) survived fewer days compared to those that received the vNDV two days prior to the HPAIV infection. Moreover, reduced transmission of vNDV to naïve contact ducks was reported. This confirms that infection with one virus can interfere with the replication of another virus, affecting its pathogenesis and transmission [57]. 

#### 2.3.4. lNDV and LPAIV in Duck

An experimental study was carried out to investigate the effect of coinfection with lNDV (Mallard/US(MN)/AI06-978/2006) and LPAIV (A/Mallard/MN/199106/99(H3N8)), in which one-monthold mallards were inoculated with lNDV and LPAIV simultaneously on the same day or sequentially two or 5 days apart. 

Co-infection revealed less productive lNDV shedding and higher LPAIV shedding in cloaca swabs. Minimal effects were observed with lNDV and LPAIV co-infection, which might reflect the adaptation of both viruses in common waterfowl, and this could stabilize virus replication and transmission in wild ducks [16]. 

## 3. Avian Influenza and Other Bacterial Co-Infections

### 3.1. Avian Influenza Coinfection with Staphylococcus sp.

*Staphylococcus* is a gram-positive bacterium that affects a wide range of avian species and widely spreads in poultry rearing environments, and presents itself as a part of normal flora of mucous membranes.

*Staphylococcus* has several pathogenicity markers, including a clumping factor that correlates with clinical cases of *Staphylococcu*s in avian species. Protein A, present on the bacterial cell surface, can bind to the fragment crystallizable region (FC) fragment of immunoglobulin and subsequently inhibit the phagocytosis of the bacteria. 

Regarding the coinfection with AIVs, *staphylococcus sp.* produces soluble proteases that are able to activate the HA of AIVs [30]. In the same context, with *in vitro* treatment of AIVs with *staphylococcus* aureus proteases, the infectivity of most strains is enhanced by at least 100 fold [58]. Furthermore, indirect activation of the HA could occur by staphylokinase that could activate chicken plasminogen into plasmin [59]. Experimentally, pre-infection of chickens with *S. aureus,* three days before infection with LPAIV H9N2 (A/chicken/aq-Y-55/01 and A/chicken/Beijing/2/97), leads to severe clinical signs. Viruses were recovered from the blood of all co-infected chickens with extensive replication in respiratory tissue, which may explain the dissemination of the virus through the chicken body and the observed severe clinical signs. In contrast, chickens infected with same LPAIV H9N2 strains alone showed no clinical signs, no virus was recovered from their blood, and there was lower replication efficiency in respiratory tissues [25]. Under field conditions, *staphylococcus sp.* are commonly recovered from chicken’s respiratory tract tissues with respiratory clinical symptoms, with a higher prevalence among other causative agents (50.2% as reported by Türkyilmaz [60] and 41.4% as reported by Popy, et al. [61]).

### 3.2. Avian Influenza Coinfection with Ornithobacterium rhinotracheale

*Ornithobacterium rhinotracheale* (ORT) is gram negative, non-motile, rod shaped, non-sporulating bacteria. ORT is reported in many countries worldwide as being associated with respiratory signs and is isolated from a wide variety of hosts such as pheasant, pigeon, rook, duck, ostrich, goose, guinea fowl, turkey, chicken, red-legged partridge, and falcon; in particular, in chickens and turkeys it causes airsacculitis and pneumonia [62,63,64,65,66,67,68]. The pathogenicity of the ORT depends on the route of inoculation, virulence of the strain, environmental factors, the immune status of the host, and the presence of the concurrent infection [62]. ORT spreads horizontally through aerosols and drinking water with longer survival rates at lower temperature, which also explains its dissemination during winter months and concurrent infection with other respiratory diseases common during winter season [69]. In China, 83% of serum collected from birds with respiratory manifestation were seropositive for ORT, and 15% of serum collected from apparent healthy birds were seropositive for ORT; five of six ORT strains recovered were associated with LPAIV H9N2 infection [70].

Furthermore, experimental preinfection with ORT and secondary infection with LPAIV H9N2 (A/chicken/Shandong/2011 (H9N2)) three days later induced the highest mortality rate with development of severe pneumonia and airsacculitis. On the other hand, a lower mortality was induced by coinfection and pre-infection with LPAIV H9N2 than in association with a secondary infection of ORT [70]. Lower mortality rate was also recorded by Azizpour*, et al.* [71] with coinfection of ORT and LPAIV H9N2 but with a different route of inoculation. Altogether, ORT as a preinfection, concurrent infection, or secondary infection is able to exacerbate the virulence of LPAIV H9N2 as compared to infection with LPAIV H9N2 alone. However, pre-infection with ORT and secondary infection with H9N2 induces a higher mortality rate with unique histopathological lesions represented by severe pulmonary fibrosis [70].

### 3.3. Avian Influenza Coinfection with Avian Mycoplasmosis 

Mycoplasmas are prokaryote that are characterized by its very small size, absence of cell wall, small genome, and being surrounded by plasma membrane. Almost 25 mycoplasma species have been isolated from various species of birds [72]. Some mycoplasma species are able to penetrate the host cells as *Mycoplasma gallisepticum* (MG) and *Mycoplasma synoviae* (MS), and these two species are also able to hemagglutinate chickens and turkeys’ RBCs [73]. MG and MS can display little to no clinical signs in avian species unless they are complicated by other respiratory pathogens [74]. Stipkovits et al. [75] demonstrated that chickens pre-infected with MG aerosol one week before challenge with LPAIV H3N8 (A/mallard/Hungary/19616/07) exhibit clinical signs and pathological lesions along the entire respiratory system (tracheitis, bronchitis, airsacculitis, and pneumonia), as well as a significant reduction in body weight gain comparing to MG alone, while LPAIV H3N8 infection did not induce clinical signs or result in a reduction in body weight gain. Furthermore, pre-infection with MG accompanied with post challenge of a LPAIV H3N8 revealed a decrease in the anti-MG antibodies level as compared to the chicken group infected with MG only [13]. 

Additionally, the host pathogen-pathogen interaction of MG and another LPAIV candidate like H9N2 (A/chicken/Saudi Arabia/CP7/1998) was also investigated using tracheal organ cultures (TOC) model as described by Sid et al., 2016 [26]. Results revealed that MG can modify the pathogenesis of LPAIV H9N2 depending on the interval between the two infections. The longer time of incubation with MG before the LPAIV H9N2 challenge enhanced ciliostasis and significant down regulated the antiviral innate immune response that subsequently enhanced the effect of H9N2. In contrast, incubation with MG for 24 h followed by a LPAIV H9N2 infection promotes both viral and bacterial replications, while longer incubation promotes only bacterial growth and viral replication, which are significantly decreased as compared to LPAIV H9N2 infection alone. This could be attributed to MG-inducing destruction of cilia, hyperplesia of epithelial cells, and desialytion of the tracheal epithelial cells. These findings could explain the ability of subclinical MG infection in birds, under field conditions, to develop severe respiratory signs with other concurrent live respiratory vaccines. These infections could be attributed to the immune modulation mediated by MG [26]. A high prevalence of LPAIV and MG and/or MS coinfection has been frequently reported in intensified poultry production areas with respiratory manifestation [76,77] that increases the severity of the clinical signs with fibro necrotic cast in the tracheal bifurcation [78].

### 3.4. Avian Influenza Coinfection with Avian Collibacillosis 

*Escherichia coli* (*E. coli*) is a gram-negative, non-spore-forming, rod-shaped bacteria that grows both aerobically and anaerobically, and many strains are motile and have peritrichous flagella. 

*E. coli* is a ubiquitous micro-organism that is wildly spread in poultry environments and is a normal inhabitant in poultry microflora. Some *E. coli* strains such as avian pathogenic *E. coli* (APEC) cause collibacillosis that could be either systemic or localized. It is widely accepted that Collibacillosis is the most common infectious bacterial diseases of poultry of all ages [79]. 

Pre-infection of chickens with LPAIV H9N2 and secondary inoculation with *E. coli* four days later was reported to induce significantly higher AIV antibodies 2 weeks post-infection (wpi) compared to secondary infection with IB or ORT. Furthermore, in the pre-infected group, a prolonged virus shedding up to 14 dpi was observed as compared to only 7 dpi in the group inoculated with H9N2 alone [80]. 

Chickens pre-infected with LPAIV H9N2 before being inoculated intrathoracically with 1.6 × 10^9^ cfu/ bird of *E. Coli (*Bekaa Valley of the Lebanon (BVL-strain) three days later showed significant early mortality with more predominant clinical signs (conjunctivitis, diarrhea, ocular exudates, and rales) and gross lesions (abdominal airsacculitis, left thoracic airsacculitis, pericarditis, right thoracic airsacculitis and tracheitis) compared to groups that received lower *E. coli* count in the challenge [81]. 

## 4. Perspectives and Future Directions

The articles discussed in this review recapitulate the adverse impacts of co/secondary viral and/or bacterial infections on AIVs infection in poultry, as well as the synergy between different pathogens (Table 1). Moreover, this review provides important insights into the variation in the rates of severe morbidity and/or mortality that subsequently occur in the case of co-infection or pre-infection with another bacterial or viral pathogen. Coinfection with AIVs and a bacterial pathogen can exacerbate the course of the viral or bacterial disease.

It has also been documented that AI-related bacterial and viral infections overall may account for up to a remarkable percent of reported cases under field conditions in different countries. In developing countries, where less biosafety measures are applied, this percentage is much higher, leading to severe economic losses in the poultry industry. This could also blur syndromic surveillance. We recommend the diagnosis of more pathogens during the inspection of an infected poultry flock that could be varied due to co-infection or pre-infection history. Moreover, the application of good biosafety and biosecurity measures is likely to reduce the severity of co-infection, and can restrict the widespread transmission of those bacterial and viral pathogens. In conclusion, this review may hopefully contribute to future knowledge regarding the diagnosis and control of avian disease among different poultry sectors.

## Figures and Tables

**Table 1:** Summary of the impact of co-infection/interference of different bacterial and viral pathogens on avian influenza viruses.

Pathogen	Strain Name	Impact	Reference
LPAI (H9N2)	A/Chicken/HK/G9/97	Had a role in protecting chickens from lethal challenge of HPAIV H5N1 infection with mild clinical sign include sneezing, nasal discharge, and ruffled feather with higher shedding in fecal material comparied to tracheal.	[36]
	Israeli LPAIV H9N2 genotype G1	Some Israeli strains showed up to 100% survival after inoculation with lethal H5N1 virus while others not.	[37]
	Egyptian LPAIV H9N2	The Egyptian LPAI H9N2 virus infection can modulate the course of subsequent infection with HPAIV H5N1.	[38]
IB	H120	Increase the severity of LPAIV H9N2 (A/chicken/Iran/SH-110/99) clinical signs that include depression, ruffled feathers, respiratory distress, swelling of the periorbital tissues and sinuses, and conjunctivitis, and nasal and ocular discharge), as well as gross lesions (as tracheal congestion, lung hyperemia and exudation of the trachea with tubular cast formation in the tracheal bifurcation). In addition, this led to a prolonged shedding period of LPAIV H9N2. It even can cause death of the infected birds.	[14]
	IBV/4/91	Chickens, co-infected simultaneously with LPAIV H9N2 (A/Chicken/Iran/SH110/99) showed severe clinical signs (respiratory distress, facial edema, conjunctivitis, depression, lacrimation, ruffled feathers, whitish watery diarrhea, and nasal discharge), gross lesions (tracheal congestion, air saculitis, lung hyperemia, tubular cast formation in the tracheal bifurcation which extended to the lower bronchi, swollen kidney, and hemorrhagic pancreas and intestine), and mortality rate (5%) with significant higher HI titer.	[47]
NDV	lNDV (LaSota)	Co-infection with LPAIV-H9N2 (A/Chicken/Pakistan/UDL/08) in chickens showed a significant reduction in virus replication and virus shedding compared to singly inoculated chickens. Further significantly higher titer for LPAIV and lower for lNDV was detected in oropharangeal and cloacal swabs, and no clinical signs were observed.	[54]
	lNDV (LaSota)	Co-infection with LPAIV H7N2 (A/turkey/VA/SEP/67/2002) induced no clinical signs, and significantly higher titer for LPAIV and lower for lNDV was detected in oropharangeal and cloacal swabs, and no clinical signs were observed.	[55]
	virulent NDV (velogenic CA/2002)	Co-infection with HPAIV (A/chicken/Queretaro/14588-19/95(H5N2) lead to reduction in the number of birds shedding HPAI. However, the death of all birds was recorded within 1.9 to 5.2 day with no difference in the clinical signs between the single infected or co-infected group in high dose of vNDV; a low dose of vNDV increased the survival of the co-infected chickens. Meanwhile, chickens infected with the less virulent mesogenic NDV prior to HPAIV (with a lower dose) revealed reduced HPAIV replication and increased survival rates.	[56]
	vNDV (APMV-1/duck/Vietnam (Long Bien)/78/2002)	Co-infection with LPAIV (A/Mallard/OH/421/1987 (H7N8)) caused no clinical signs in ducks. However, co-infection decreased the number of birds shedding vNDV, and it did not affect the number of ducks shedding LPAIV (except at 2 dpi low virus shedding was observed in the co-infected group than the group infected only with LPAI). HPAI (A/duck/VN/NCVD-672/2011 (H5N1)) survived fewer days compared to those that received the vNDV two days prior the HPAIV. Moreover, reduced transmission of vNDV to naı¨ve contact ducks was reported.	[57]
	lNDV Mallard/US(MN)/AI06-978/2006	Co-infection with LPAIV (A/Mallard/MN/199106/99 (H3N8)) revealed less productive lNDV shedding and higher LPAIV shedding and in the cloaca swabs minimal effects were observed with lNDV and LPAIV co-infections.	[16]
*Staphylococcu*s sp.	*S. aureus*	Experimentally, pre-infection of chickens with S. aureus, 3 days before infection with LPAIV H9N2 (A/chicken/aq-Y-55/01 and A/chicken/Beijing/2/97 ), lead to severe clinical signs. Viruses recovered from blood of all co-infected chickens with extensive replication in the respiratory tissue.	[25]
Ornithobacteriumrhinotracheale (ORT)	ORT/chicken/ Shandong/2011	Experimental preinfection with ORT and 3 days later secondary infection with LPAIV H9N2 (H9N2/chicken/Shandong/2011) induced the highest mortality rate with development of severe pneumonia and airsacculitis with unique histopathological lesions represented by severe pulmonary fibrosis. On the other hand, a lower mortality rate induced by coinfection and pre-infection with LPAIV H9N2 then secondary infection with ORT.	[70]
Avian mycoplasmosis	Strain 122610	Chickens pre-infected with MG aerosol one week before challenge with LPAIV H3N8 (A/mallard/Hungary/19616/07) exhibit clinical signs, pathological lesions along the entire respiratory system (tracheitis, bronchitis, airsacculitis and pneumonia) as well as reduction in body weight gain significantly comparing to single infection with decrease in the anti-MG antibodies level comparing to group infected with MG only.	[13,75]
	MG S6 lab. strain	MG modifies the pathogenesis of LPAIV H9N2 (A/chicken/Saudi Arabia/CP7/1998) using tracheal organ cultures (TOC) model depending on the interval between the two infections.The longer time incubation with MG before LPAIV H9N2 challenge the more enhancement of ciliostasis and significant down regulation of antiviral innate immune response that subsequently enhances the effect of H9N2. While longer incubation promote only bacterial growth while viral replication significantly decreased.	[26]
*Escherichia coli*		Pre-infection of chickens with LPAIV H9N2 (A/chicken/Pakistan/31/01) and 4 days later secondary inoculated with *E. coli* induce higher AIV antibodies at 2 wpi comparing to secondary infection with IB or ORT. Furthermore, In the pre-infected group, a prolonged virus shedding up to 14 dpi compared to only 7 dpi in the group inoculated with H9N2 alone was recorded.	[80]
	Bekaa Valley of the Lebanon (BVL-strain)	Chickens pre-infected with LPAIV H9N2 then 3 days later inoculated with 1.6 × 109 cfu/ birds of E. Coli intrathoracic showed significant early mortality with more predominant clinical signs (conjunctivitis, diarrhea, ocular exudates and rales) and gross lesion (abdominal airsacculitis, left thoracic airsacculitis, pericarditis, right thoracic airsacculitis and tracheitis) comparing to groups that received lower *E. coli* count used in the challenge	[81]
